# Treatment of Haemophilia by Injections of Serum

**Published:** 1907-02

**Authors:** 


### TREATMENT OF HAEMOPHILIA BY INJECTIONS OF SERUM

---

Weil (*La Tribune medicale,* November 10, 1906) report successful results in the treatment of haemophilia by injections of serum, either human, or obtained from the horse or ox. He distinguishes two forms of haemophilia, the spontaneous and the hereditary, or family type. The former is less marked in its manifestations, either in frequency or gravity, than is the latter. It appears in these acquired cases that a trauma of some importance is needed to produce the obstinate haemorrhage. The recurrence of the bleedings may be separated for several years, and the general health may be excellent. In the hereditary type, on the contrary, and every lesion is followed by prolonged haemorrhage. Epistaxis and viscerial bleedings are of common occurrence, often without apparent cause. Effusions of blood into the joints may follow violent exercises, or fatigue, and lead to permanent lameness. The general health is affected. In the spontaneous or acquired form a study of the blood appears to show that it has undergone no pathological medification execpt theabsence or an alteration of the kinase. In the inherited form, the cause of the incoagulability of the blood is much more complex. There is some evidence that it is due in part to the presence of some substance which delays coagulation. This may possibly be of hepatic origin. It was found, however, that by the injection of fresh serum the tendency to bleeding was greatly reduced in this class of patients, and entirely overcome in the cases of acquired haemophilia. The injections of fresh serum were given in doses usually of from ten to twenty cubic centimetres. Human serum or serum from the horse was found to be better borne than the bovine serum. After administering the latter, almost immediately as a rule there was a short but violent febrile attack, with chilly sensations, yawning, vomiting, and pain in the back. A single injection in a case of spontaneous or acquired haemophilia was found to be sufficient to restore the blood for the time to its integrity. The immunity for bleeding continues for two weeks to a month. The injections may be repeated if thought necessary at the end of this period. The serum injection does not act upon the cause of the condition, but is merely a symptomatic treatment. The method, however, is very useful as a prophylactic in a patient, known to be a bleeder upon whom a surgical operation may be required, or even for the extraction of teeth. It is also curative in cases of haematuria and other conditions of prolonged bleeding. As pointed out by Widal and Courmont, the serum from the horse may be injected into a vein without any bad result, and this is also true of human serum.—*N. Y. Medical Journal, Jan.* 5, '07.

